# Commentary: Portuguese crypto-Jews: the genetic heritage of a complex history

**DOI:** 10.3389/fgene.2015.00261

**Published:** 2015-08-07

**Authors:** Alexander W. Marcus, Emily R. Ebel, Daniel A. Friedman

**Affiliations:** ^1^Department of Religious Studies, Stanford UniversityStanford, CA, USA; ^2^Department of Biology, Stanford UniversityStanford, CA, USA

**Keywords:** Jewish genetics, haplotypes, crypto-Jews, Iberian Peninsula, admixture, demographic history

## Introduction

Nogueiro et al. (2015) utilize Y chromosome and mitochondrial genotype data from a contemporary Iberian and non-Iberian human populations to explore the genetic identity of Portuguese “crypto-Jews.” In the first section of the paper, a historical introduction reviews the plight of Jews in the Iberian Peninsula from the earliest archaeological evidence, through the Inquisition, to the current day. In the second section, Y chromosome and mtDNA population genetic data from many previous studies are reviewed, along with a reanalysis of data from two Portuguese Jewish populations. In the final section, a historical narrative of the Portuguese crypto-Jews is presented, caveats to the analysis are discussed, and future directions are suggested.

## Critique 1

Many of the data reviewed suffer from methodological issues that weaken or invalidate the conclusions made. Specifically, the data are ambiguous with regards to the inference of Jewish ancestry and do not identify diagnostic patrilineal or matrilineal markers.

## Critique 2

The authors suggest that the data may support a history of “complex mating strategies” in Jewish populations, but fail to test this hypothesis against models of even simple demographic histories or admixture events. Without consideration of alternative hypotheses, it is not possible to draw inferences about how the crypto-Jews must have “belong[ed] to a distinctive community,” especially with regards to belief and practice.

## Explanation of critique 1

The analysis of Nogueiro et al. (2015) uses previous work on uniparental markers (Y chromosomal and mtDNA) to explore the possibility of a diagnostic genetic pattern of Jewish ancestry. We have reservations about the strength of the matrilineal (mtDNA) and patrilineal (Y chromosomal) data reviewed and presented in this analysis. A recent study (Tofanelli et al., 2014) finds that Jewish haplotype motifs in much previous work are inadequate for “forensic or genealogical purposes,” because ambiguity in the molecular clock, along with haplotype polyphyly, preclude their usage as “reliable Jewish ancestry predictors.” Furthermore, the cited studies do not, even amongst themselves, find a diagnostic Jewish “genetic profile.” Many of the “Jewish” haplotypes cited by Nogueiro et al. (Gonçalves et al., 2005; Pacheco et al., 2005) are pan-Middle Eastern markers—common in self-identified Jews, but also in multiple Arabic lineages. Thus, these haplotypes are of ambiguous ancestry—they could be of Jewish ancestry, Arab ancestry, or observed at high frequencies due to more complex demographic scenarios.

Nogueiro et al. acknowledge that “the inference of a genetic profile for the Portuguese Jews was not possible” from a study of self-defined Sephardic Jews (Adams et al., 2008), and instead focus their analysis on two smaller Jewish populations. Their acknowledgment that there is no diagnostic Jewish genetic profile, which is supported by the data presented, appears to contradict the major conclusion of the paper: that Iberian Jews have “succeeded in maintaining a genetic heritage of their own.”

## Explanation of critique 2

To answer questions of medical and cultural relevance, population-level genetic analyses must carefully control for population structure using complex statistical methodologies on high-quality, whole-genome scale datasets (Pickrell and Pritchard, 2012). This is to ensure that observed genetic patterns are not merely false-positives stemming from situations where human demography violates certain simple assumptions (Liu et al., 2013). Admixture is one of the central forces in shaping global patterns of human genetic diversity, and is difficult to estimate with confidence (Hellenthal et al., 2014). Even in very recent admixture events where whole-genome data is available, findings can be ambiguous (Jin et al., 2012). Small sample sizes of uniparental markers, as used in Nogueiro et al. (2015), are unable to draw robust conclusions.

Inferring historical admixture patterns in contemporary populations is a quantitative, empirical question. However, the analysis of Nogueiro et al. rests on qualitative patterns of diversity, which are not tested against any models of human demography. For example, patterns of diversity in both mitochondrial and Y-chromosome haplotypes in the Bragança Jews more closely resemble patterns in the Portuguese population than in the Belmonte Jews. Although Nogueiro et al. call this “extremely surprising,” given their expectation of severe inbreeding, they attribute it to “complex mating strategies and/or a very heterogeneous genetic pool in their origin.” These findings are “extremely surprising” only when they are “translated in a conscience of belonging to a distinctive community”—the author's pre-existing model of crypto-Jew reproductive isolation.

Hypotheses of admixture in the Bragança Jews and a bottleneck in the Belmonte Jews could be statistically evaluated using extant demographic models. Together with historical data, quantitative estimates of admixture could be used to infer the interesting and potentially unique histories of these populations. However, this opportunity is missed by focusing primarily on diagnosing a Jewish genetic signature.

## Conclusion

Nogueiro et al. (2015) interpret many ambiguous and contradictory population genetic findings within an *a priori* model of Portuguese crypto-Jew reproductive isolation. The results and analysis offer no concrete support for specific statements about the mating habits, beliefs, and/or praxis of these historical Jewish populations. Our critique is consistent with the previously-recognized difficulty of making claims about both Jewish haplotype motifs and late-antique and early-medieval Iberia (Astren, 2004; Tofanelli et al., 2014; Falk, 2015). Textual and material evidence dating from the period following the Inquisition provide a complex picture of both inter-marriage and communal isolation in the Iberian Peninsula (Nirenberg, 2014). Given the synchronic and diachronic complexities of Iberian population dynamics, as well as the ambiguous interpretation of molecular markers, the claim of the persistence of “crypto-Judaism”—a secret adherence to Jewish belief and practice across generations—is one that requires more careful examination. Especially when statements are of political or cultural relevance, human population genetic researchers must be careful to draw only data-supported conclusions.

## Funding

This work was supported by a National Science Foundation Graduate Research Fellowship to EE (DGE-1247312) and an Urbanek Family Stanford Graduate Fellowship to DF.

### Conflict of interest statement

The authors declare that the research was conducted in the absence of any commercial or financial relationships that could be construed as a potential conflict of interest.
